# Correlations of sST2 and Gal-3 with Cardiothoracic Ratio in Patients with Chronic Kidney Disease

**DOI:** 10.3390/biomedicines12040791

**Published:** 2024-04-03

**Authors:** Ying-Ju Chen, Che-Yi Chou, Tze-Kiong Er

**Affiliations:** 1 Division of Laboratory Medicine, Asia University Hospital, Asia University, Taichung 41354, Taiwan; 2 Division of Nephrology, Asia University, Taichung 41354, Taiwan; 3 Department of Medical Laboratory Science and Biotechnology, Asia University, Taichung 41354, Taiwan; 4 Department of Nursing, Asia University, Taichung 41354, Taiwan

**Keywords:** soluble ST2, Galectin-3, cardiothoracic ratio, chronic kidney disease, cardiovascular disease

## Abstract

Chronic kidney disease (CKD) frequently correlates with cardiovascular complications. Soluble suppression of tumorigenicity 2 (sST2) and Galectin-3 (Gal-3) are emerging as cardiac markers with potential relevance in cardiovascular risk prediction. The cardiothoracic ratio (CTR), a metric easily obtainable from chest radiographs, has traditionally been used to assess cardiac size and the potential for cardiomegaly. Understanding the correlation between these cardiac markers and the cardiothoracic ratio (CTR) could provide valuable insights into the cardiovascular prognosis of CKD patients. This study aimed to explore the relationship between sST2, Gal-3, and the CTR in individuals with CKD. Plasma concentrations of sST2 and Gal-3 were assessed in a cohort of 123 CKD patients by enzyme-linked immunosorbent assay (ELISA). On a posterior-to-anterior chest X-ray view, the CTR was determined by comparing the widths of the heart to that of the thorax. The mean concentration of sST2 in the study participants ranged from 775.4 to 4475.6 pg/mL, and the mean concentration of Gal-3 ranged from 4.7 to 9796.0 ng/mL. Significant positive correlations were observed between sST2 and the CTR (r = 0.291, *p* < 0.001) and between Gal-3 and the CTR (r = 0.230, *p* < 0.01). Our findings indicate that elevated levels of sST2 and Gal-3 are associated with an increased CTR in CKD patients. This relationship may enable better cardiovascular risk evaluation for CKD patients. Further studies are warranted to explore the clinical implications of these associations.

## 1. Introduction

Chronic kidney disease (CKD) is characterized by a gradual loss of kidney function, eventually progressing to end-stage renal disease (ESRD). As the disease becomes more chronic, complications such as anemia, electrolyte disturbances, mineral disorders, and atherosclerosis become increasingly probable. Such complications precipitate systemic alterations, notably impacting cardiac function. Both kidney disease and hemodialysis (HD) significantly amplify the disturbances in cardiac activities, leading to conditions such as cardiomyopathy, diastolic dysfunction, congestive heart failure, coronary heart disease, and vascular modifications [1]. In both CKD and non-CKD populations, established and emerging cardiac biomarkers have been identified as linked to heart failure (HF) risk [2]. Of the biomarkers under study, growth/differentiation factor-15 (GDF-15), Galectin-3 (Gal-3), and soluble suppression of tumorigenicity 2 (sST-2) stand out as potential key markers for cardiovascular disease (CVD) and its outcomes, possibly offering insights into the cardiac structure [3]. They correlate with significant cardiovascular events in the broader population [4,5,6] and more recently, in CKD patients [7]. Therefore, identifying and validating particular cardiac markers indicative of CVD-associated risks will be essential. In our latest review article, we explored the link between cardiovascular biomarkers and treatment approaches for CKD in determining patient outcomes, underscoring the importance of the former. The identification and validation of specific cardiac indicators linked to CVD risks will be essential [8].

In the ongoing pursuit of improving cardiovascular risk stratification in CKD patients, the quest to identify markers that can provide enhanced insights continues. Among the notable biomarkers garnering significant interest are sST2 and Gal-3. sST2, part of the interleukin-1 receptor family, is acknowledged as a crucial indicator of myocardial stretch and fibrosis [9]. ST2 was first discovered in 1989 as a factor involved in inflammatory and autoimmune diseases [10,11]. The interaction between ST2 and its ligand, IL-33, plays a crucial role in modulating the immune response. Elevated levels of sST2 in the blood have been observed in conditions such as asthma, septic shock, trauma, and systemic lupus erythematosus [12], not only in inflammatory conditions but also across a spectrum of cardiac disorders, where they act as a crucial prognostic marker for CVD. [13]. Further, sST2 plays a pivotal role as a prognostic marker in determining the severity and mortality risk associated with cardiovascular diseases. Its effectiveness in predicting mortality extends to patients with conditions like stable coronary artery disease and chronic heart failure, underlining the value of sST2 in the assessment of cardiovascular risk and patient prognosis [14]. sST2 is also linked to inflammation in myocardial infarction (MI) and HF, indicating its role in the pathophysiology of these cardiac conditions [15]. Recent studies have frequently reported an association between sST2 and CVD, particularly highlighting its relevance in HF. A meta-analysis examining serum sST2 levels in different cardiovascular conditions found that individuals with HF had notably higher levels than those without CVD [16]. Patients with stable HF typically exhibit low levels of soluble suppression of sST2. However, in acute heart failure (AHF), elevated sST2 levels in the plasma are of prognostic significance [17,18]. Aimo A. et al. demonstrated that sST2 concentrations were predictive of overall mortality, cardiovascular death, and a combined endpoint of death from any cause or hospitalization due to heart failure in patients with AHF. Notably, the prognostic value was not limited to the sST2 levels at admission; the levels at discharge also served as predictors for the likelihood of readmission due to heart failure [18]. Thus, sST2 holds significant clinical value for managing CVD in patients with concurrent CKD.

Gal-3, a component of the lectin family, serves as a galactoside-binding protein, crucial for several biological activities. It facilitates interactions between cells and their matrix, controlling cellular adhesion and proliferation, affecting the cell death processes, engaging in pre-mRNA splicing, and influencing immune and inflammatory reactions [19,20]. Additionally, Gal-3 plays a pivotal role in the regulation of inflammation and associated conditions, with its proinflammatory effects tied to the activation of the NF-kB transcription factor, the induction of tumor necrosis factor α (TNF-α) and interleukin 6, the management of cell adhesion, the enhancement of cell activation and movement, and the regulation of cellular growth or programmed cell death [21]. In a rat model post-myocardial infarction, an increase in Gal-3 levels was observed, with a delayed peak occurring in the non-infarcted myocardium, indicating its involvement in cardiac remodeling. [22]. A comprehensive review by Hara A. et al. highlighted the potential of Gal-3 as a diagnostic marker for detecting the early stages of various heart diseases, potentially enhancing the effectiveness of early therapeutic interventions [23]. Gal-3 also plays a key role in the pathophysiology of HF, primarily due to its involvement in cardiac ventricular remodeling [24,25]. Under normal conditions, Gal-3 expression in the heart is minimal, but its production and release are elevated in the context of HF [26]. Further, a recent meta-analysis by Cheng Z. et al. unveiled the prognostic significance of serum Gal-3 for both all-cause mortality and cardiovascular-related deaths among patients with chronic HF, highlighting its potential as a valuable tool for risk stratification in these patients [27]. Additionally, a study on the readmission rates of HF patients found that individuals with Gal-3 levels exceeding 17.8 ng/mL had a notably higher rate of rehospitalization. This finding indicates that serum Gal-3 levels serve as a predictive marker for the likelihood of near-term readmission in patients hospitalized for heart failure [28]. In the realm of cardiovascular diseases, Gal-3 contributes significantly to the development of fibrosis, the progression of atherosclerosis, and the broader spectrum of inflammation-based conditions. A key feature of Gal-3 is its utility as a biomarker for cardiovascular disease risk when its levels are assessed in plasma [29]. Taken together, sST2 and Gal-3 are superior to other cardiac biomarkers because they specifically indicate cardiac stress and fibrosis, providing unique insights into heart disease progression. Their ability to predict adverse cardiovascular outcomes has been consistently demonstrated across various patient cohorts, making them more effective for early detection and risk assessment.

Another parameter of interest, especially in limited resource settings, is the CTR. Traditionally determined using chest radiographs, the CTR provides an estimate of cardiac size and has been correlated with left ventricular hypertrophy, left ventricular mass, and HF [30,31]. A previous study has highlighted that a CTR exceeding 55% is a notable independent predictor of all-cause mortality over two years, indicating the potential benefit in early cardiac status assessment using CTR in patients commencing HD, to potentially improve survival outcomes [32]. In support of this, Ogata H. and colleagues showed that the CTR, in conjunction with various clinical parameters, can significantly forecast all-cause and cardiovascular mortality, along with a range of composite outcomes in individuals receiving HD [33]. Despite these associations, the specific interplay between the CTR and the cardiac biomarkers soluble suppression of sST2 and Gal-3 within the CKD patient cohort remains underexplored.

In this study, we investigate the correlation between sST2, Gal-3, and the CTR in patients with CKD, hypothesizing that increased levels of these cardiac markers may be associated with an altered CTR, thus providing insights into the cardiovascular changes in this patient population.

## 2. Materials and Methods

### 2.1. Study Design and Participants

This cross-sectional study encompassed 123 patients diagnosed with stage 5 CKD, all of them undergoing maintenance dialysis, either through hemodialysis or peritoneal dialysis, from August 2022 to November 2022. The chosen sample size was meticulously calculated based on a power analysis conducted in the initial phase of the study planning. This study had adequate statistical power to identify a significant correlation between the biomarkers sST2, Gal-3, and the CTR in the CKD patient group.

Criteria for inclusion in the study were specifically designed to include individuals aged 18 and older who had been receiving dialysis for stage 5 CKD for more than three months and were capable of providing informed consent. Before participation, written informed consent was secured from all participants, affirming their voluntary engagement and understanding of the study’s objectives and procedures.

Ethical considerations were stringently observed, with the study receiving formal approval from the Institution Review Board of the China Medical University Hospital (CMUH111-REC3-105). The ethical guidelines of this research adhered strictly to the internationally recognized Declaration of Helsinki, which sets forth the ethical principles for medical research involving human subjects. The study also complied with the principles of Good Clinical Practice.

### 2.2. Sample Collection

Blood samples were meticulously collected under controlled and stable conditions to ensure the integrity of the specimens. Once collected, these samples were immediately subjected to centrifugation at a force of 1000 g for 15 min. Following centrifugation, the plasma layer was carefully extracted and transferred to sterile, labeled vials. To preserve the biochemical stability of the samples and prevent the degradation of sensitive biomarkers, they were promptly frozen and stored at a temperature of −80 °C, ensuring that the samples remained in optimal condition for subsequent analyses.

### 2.3. Measurement of sST2 and Gal-3

The plasma samples were analyzed for sST2 and Gal-3 concentrations using enzyme-linked immunosorbent assay (ELISA) kits. For sST2, the Quantikine ELISA Kit (R&D Systems^®^, Minneapolis, MN, USA) was used, following the manufacturer’s instructions. The interassay and intra-assay coefficients of variation were 5.4% and 4.4%, respectively. Similarly, Gal-3 levels were determined using the Quantikine ELISA Kit (R&D Systems^®^). The interassay and intra-assay coefficient of variation were 5.8% and 3.5%, respectively. Absorbance was read on the infinite^®^ F50 ELISA reader platform at 450 nm, and concentrations were derived from standard curves generated for each assay.

The standard curves of sST2 were in the concentration range 31.3–2000 pg/mL. To achieve a 20-fold dilution of plasma samples, it is recommended to mix 10 μL of the sample with 190 μL of Calibrator Diluent RD5-26, which had been diluted at a 1:4 ratio. To each well was added 50 μL of Assay Diluent RD1-63 and 50 μL of standard, control, or sample. The plate was first incubated at room temperature for 2 h. After a wash, 100 μL of horseradish peroxidase-conjugated antibody reagent was added and incubated for another 2 h. After another washing step, 100 μL of substrate solution was added to each well and incubated in the dark for 30 min. Subsequently, 100 μL of stop solution was introduced, and the absorbance was measured at 450 nm.

The standard curves of Gal-3 were in the concentration range 0.313–10.0 ng/mL. To achieve a 2-fold dilution of plasma samples, it is recommended to combine 75 μL of the sample with 75 μL of Calibrator Diluent RD6X, which had been diluted at a 1:5 ratio. To each well was added 100 μL Assay Diluent RD1W and 50 μL of standard, control, or sample were combined. The plate was initially incubated at room temperature for 2 h. Following a wash, 200 μL of horseradish peroxidase-conjugated antibody reagent was added and incubated for an additional 2 h. After another washing step, 200 μL of substrate solution was added to each well and incubated in the dark for 30 min. Subsequently, 50 μL of stop solution was introduced, and the absorbance was measured at 450 nm.

### 2.4. Cardiothoracic Ratio (CTR) Assessment

The CTR is a diagnostic measure defined as the ratio between the widest parts of the heart and the chest cavity, as observed on a posterior–anterior (PA) chest radiograph. This ratio is calculated by measuring the greatest transverse dimension of the heart (A + B) and dividing it by the greatest transverse dimension of the chest cavity (C), measured to the inner surface of the ribs. This calculation is depicted in Figure S1. A CTR value exceeding 0.5 is clinically significant, indicating an enlarged heart.

### 2.5. Statistical Analysis

Data were analyzed using R software, version 4.2.1. We calculated the median and interquartile range for age and laboratory data. The gender and primary kidney disease of the patients were summarized using counts and percentages. The correlations between sST2, Gal-3, and the CTR were assessed using Pearson’s correlation analysis. Univariable and multivariable linear regression analyses were conducted to reveal the associations between sST2, Gal-3, and the CTR. We conducted multivariable linear regression adjusting for age, gender, and laboratory data. With consideration for the variable normality, we conducted a log transformation prior to conducting linear regression analysis. A *p*-value of less than 0.05 was considered to indicate statistical significance.

## 3. Results

### 3.1. Study Population

In our study, we analyzed data from 123 participants, having an average age of 64.7 years and a standard deviation of 11.8. The median age was closely aligned at 65 years, with participant ages ranging from 33 to 91. The gender composition of our sample was 59.3% male (73 participants) and 40.7% female (50 participants). Detailed demographic, laboratory, and cardiac marker data are comprehensively presented in Table 1, which encapsulates the clinical characteristics of our study population.

### 3.2. sST2 and CTR

**Figure 1:**
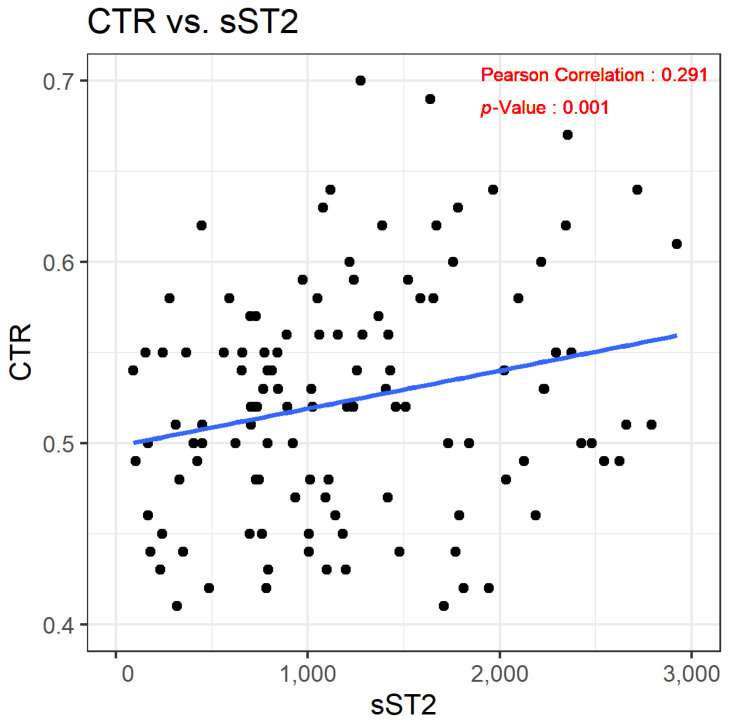
Scatterplot demonstrating the correlation between sST2 and the CTR. Relationships were evaluated using Pearson’s correlation coefficient.

In our study, the sST2 concentrations among participants varied widely, with values ranging from 775.4 to 4475.6 pg/mL, indicating a significant variability in sST2 levels within our CKD cohort. Through statistical analysis, we discovered a notable positive correlation between the levels of sST2 and the CTR. Specifically, the Pearson’s correlation coefficient (r) was calculated at 0.291, demonstrating a moderate association. The significance of this correlation was underscored by a *p*-value of less than 0.001, strongly suggesting that individuals with higher sST2 levels tend to exhibit an increased CTR. This relationship is graphically represented in Figure 1, depicting the correlation between sST2 levels and the CTR in our study’s CKD population. The univariable model revealed that an increase in sST2 levels is associated with a corresponding increase in the CTR, with a coefficient (β) of 2.27 × 10^−5^ and a *p*-value of 0.001 (Table 2).

### 3.3. Gal-3 and CTR

**Figure 2:**
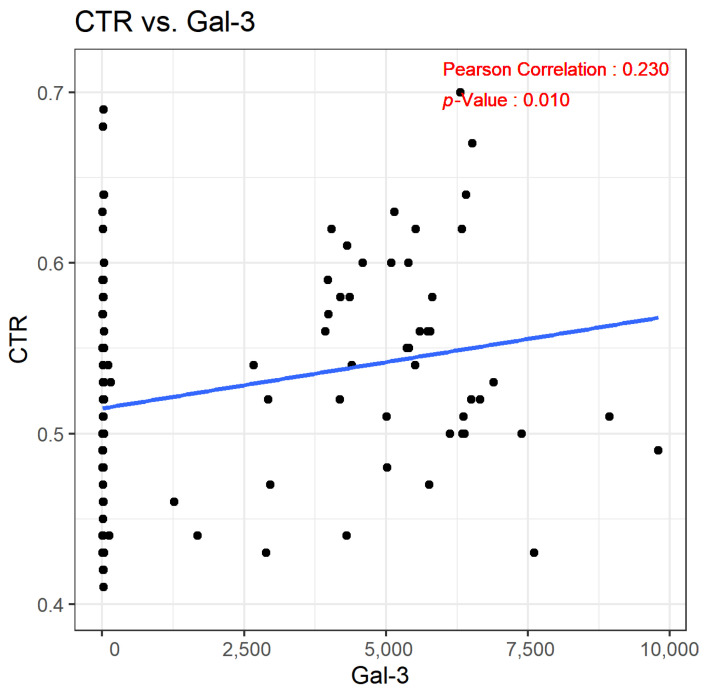
Scatterplot demonstrating the correlation between Gal-3 and the CTR. Relationships were evaluated using Pearson’s correlation coefficient.

In our analysis, the Gal-3 levels among the study participants exhibited a broad range, with the mean concentration ranging from 4.7 ng/mL to an impressive 9796.0 ng/mL. This wide range highlights the variability of Gal-3 levels in individuals with CKD. We identified a positive correlation between Gal-3 concentrations and the CTR. The statistical analysis yielded a Pearson’s correlation coefficient (r) of 0.230, indicating a mild to moderate positive relationship. This correlation was statistically significant, with a *p*-value of less than 0.01, reinforcing the notion that CKD patients with higher levels of Gal-3 are more likely to have an increased CTR. This association is visually represented and detailed in Figure 2, which illustrates the link between elevated Gal-3 levels and the CTR in the CKD patient cohort. Referring to Table 2, a positive association between Gal-3 concentrations and the CTR is found, with a coefficient (β) of 5.42 × 10^−6^ and a *p*-value of 0.011.

### 3.4. Combined Analysis

In the multivariate linear regression model, when considering both biomarkers together, sST2 and Gal-3 emerged as independent predictors of an increased CTR in our CKD cohort. The adjusted beta values were 2.00 × 10^−5^ (*p*-value = 0.006) and 5.33 × 10^−6^ (*p*-value = 0.055) for sST2 and Gal-3, respectively, as shown in Table 2. After log transformation, the adjusted beta values were 0.018 (*p*-value = 0.025) for sST2 and 0.012 (*p*-value = 0.158) for Gal-3.

## 4. Discussion

In our comprehensive analysis involving 123 patients with CKD, we uncovered compelling evidence supporting the association between two novel cardiac biomarkers, sST2 and Gal-3, and the CTR. The CTR, a conventional and easily obtainable measure, serves as an indicator of cardiac size and the potential for cardiomegaly. Traditional methods for detecting cardiac anomalies typically reveal abnormalities only at advanced stages of structural heart changes. However, our research indicates that simultaneous measurement of sST2 and Gal-3 with the CTR can facilitate the early identification of CKD patients at an increased risk of cardiac alterations, even before such changes become detectable through standard imaging techniques. This integrative approach enhances the capacity for early diagnosis, enabling the initiation of timely medical interventions that could potentially slow down or arrest the progression of CVD in CKD patients. Ultimately, our research highlights the significance of integrating these novel cardiac markers with conventional imaging techniques, setting the stage for enhanced cardiovascular risk evaluation and management among CKD patients.

While advanced cardiac imaging techniques provide comprehensive insights, they might not be universally accessible, especially in limited-resource settings. The CTR, a parameter derived from basic chest radiographs, when combined with the assessment of the cardiac biomarkers, offers a cost-effective and widely accessible method for cardiovascular risk assessment in CKD patients. When the CTR was initially developed, and for many years of its application, values exceeding 0.5 were viewed as indicative of both heart enlargement and compromised left and/or right ventricular function [34]. Further, elevated CTR values typically indicate a worse prognosis, with two long-term studies on healthy populations revealing strong correlations between high CTR values and cardiovascular mortality [35,36]. The CTR has been identified as a prognostic indicator across various disease categories, notably among patients receiving hemodialysis. For instance, a comprehensive, prospective cohort study involving nearly 3500 participants over four years demonstrated that among HD patients, an elevated CTR correlates with increased mortality risks, both overall and cardiovascular-specific [37]. Multiple studies demonstrated that the CTR serves as a crucial marker for mortality and declining functional health in patients initiating dialysis and those with ESRD undergoing consistent HD or peritoneal dialysis [32,37,38,39,40]. Recently, Chou et al. used a machine learning algorithm to analyze the CTR in 3117 CKD patients from Taiwan and found that higher CTR values significantly correlated with increased risks of ESRD [40]. Their findings confirm the practical prognostic value of the CTR, as assessed by a machine learning annotation tool, within the setting of CKD. Additionally, Okute Y. et al. demonstrated that in the absence of NT-proBNP, the CTR can serve as a tool for CVD risk assessment in HD patients [41]. Taken together, the CTR has proven clinical utility in assessing CVD risk among CKD patients.

The significant positive correlation observed between sST2 levels and CTR aligns with existing literature linking elevated sST2 concentrations to myocardial stress and fibrosis [16]. sST2, part of the interleukin-1 receptor family, has been previously linked with adverse cardiac outcomes, especially in the context of HF and myocardial infarction [42,43]. On the other hand, a study discovered that sST2 serves as a CKD biomarker, with its diagnostic accuracy remaining largely unaffected by reduced renal function [44]. They proposed that sST2 could be a promising biomarker for managing CVD patients. An observational case-cohort study conducted by Bansal N. et al. demonstrated that rising levels of NT-proBNP over two years were significantly linked to increased risks of both HF and atrial fibrillation, while elevated sST2 was associated with HF, suggesting potential biomarkers for cardiovascular risk in CKD patients [45]. Recently, Hammer F. et al. demonstrated that sST2 potently and independently forecasts lethal incidents in a sizable group of HD patients with type 2 diabetes mellitus [46]. Our findings suggest that in CKD patients, higher sST2 levels might indicate increased cardiac size or potential cardiomegaly, emphasizing the intricate cardiovascular implications in renal dysfunction.

The positive association between Gal-3 levels and CTR emphasizes the significance of this beta-galactoside-binding lectin, which plays a role in fibrogenesis, tissue repair, myofibroblast proliferation, and myocardial restructuring [47]. Gal-3 has been previously associated with HF and adverse cardiovascular events. Alam M.L. et al. suggested that elevated levels of Gal-3 might correlate with CKD progression, shedding light on the potential new mechanisms influencing the advancement of kidney disease. [48]. Further, the circulating level of Gal-3 has been identified as indicating potential abdominal aortic calcification in patients on HD [49]. An increase in Gal-3 levels in HD patients has also been recognized as elevating their cardiac death risk [50]. In this study, we showed that the relationship with the CTR suggests that Gal-3 might also indicate early cardiac structural changes in CKD patients.

Cardiovascular complications in patients with CKD are influenced by a range of factors, such as volume overload, hypertension, and uremic effects. Our study contributes to understanding this complexity by indicating that elevated levels of cardiac biomarkers, specifically sST2 and Gal-3, may be associated with early structural heart changes, possibly preceding the manifestation of symptoms. While our findings of a correlation between sST2, Gal-3, and the CTR in CKD patients offer insights into the potential for these biomarkers in cardiovascular risk assessment, it is important to recognize the limitations of our study. The correlations found, although statistically significant, explain only a modest portion of CTR variability, suggesting that other factors play significant roles in cardiac changes in CKD patients.

Our research, which is based on a single-time point measurement from a relatively small, single-center cohort, might not capture the full dynamics of biomarker fluctuations and their long-term effects on cardiac health. Additionally, our study faced limitations in accurately detailing dialysis vintage and medication history, due to the retrospective nature and data source constraints. This limitation underscores the necessity of interpreting our findings as preliminary, and of more extensive research to corroborate and expand upon these initial observations.

## 5. Conclusions

In conclusion, while our research indicates a potential link between sST2, Gal-3, and the CTR in CKD patients, these results should be interpreted with caution. The integration of sST2 and Gal-3 with traditional cardiac assessment tools like CTR may enhance cardiovascular risk stratification in CKD patients. However, further studies are required to validate these biomarkers’ utility and explore their role in comprehensive management strategies for this high-risk group. It is essential to develop predictive models that incorporate these biomarkers to improve risk assessment and guide therapeutic decisions. Our study underscores the continuing need to refine our understanding of cardiac biomarker dynamics in CKD, aimed at improving patient outcomes through targeted interventions.

## Figures and Tables

**Table 1:** Clinical characteristics of CKD patients.

Laboratory Data	ESRD (*n* = 123)
**Demographic data**	
Age (y)	64.7 ± 11.8
Male *n* (*n*%)	73 (58.9%)
Female *n* (*n*%)	50 (41.1%)
Patients treated with PD	49 (39.8%)
Patients treated with HD	74 (60.2%)
**Causes of CKD**	
Diabetes	39 (31.7%)
Hypertension	41 (33.3%)
Chronic glomerulonephritis	37 (30.1%)
Others	6 (4.9%)
**Laboratory data**	
Hemoglobin, g/L	10.3 ± 1.5
Creatinine, µmol/L	1131.5 ± 344.8
Albumin, g/L	37 ± 4
Calcium, mmol/L	2.275 ± 0.225
Phosphorus, mmol/L	1.87 ± 0.48
Sodium, mmol/L	136.3 ± 3.3
Potassium, mmol/L	4.2 ± 0.7
PTH intact, pmol/L	489.6 ± 389.6
CaxP mmol^2^/L^2^	53 ± 14.9
CTR	0.5 ± 0.1
**Cardiac markers**	
sST2, ng/mL	1.9861 ± 1.1522
Gal-3, ng/mL	4934.3 ± 2018.7

ESRD: end-stage renal disease, PD: peritoneal dialysis, HD: Hemodialysis, CaxP: calcium x phosphorus, CTR: cardiothoracic ratio, sST2: soluble suppression of tumorigenicity 2, Gal-3: Galectin-3.

**Table 2:** Linear regression of CTR and sST2/ Gal-3.

Variables	Univariable Model		Multivariable Model ^†^	
	β	*p*-Value	β	*p*-Value
Linear regression model				
sST2	2.27 × 10^−5^	0.001	2.02 × 10^−5^	0.007
Gal-3	5.42 × 10^−6^	0.011	4.79 × 10^−6^	0.293
Linear regression model with log transformation				
sST2	0.023	0.002	0.018	0.025
Gal-3	0.005	0.010	0.012	0.158

CTR: cardiothoracic ratio, sST2: soluble suppression of tumorigenicity 2, Gal-3: Galectin-3. ^†^: adjusted for age, gender, sST2, Gal-3, PH/HD, and laboratory data listed on Table 1.

## Data Availability

Data are contained within the article and Supplementary Materials.

## Supplementary Materials

The following supporting information can be downloaded at: https://www.mdpi.com/article/10.3390/biomedicines12040791/s1, Figure S1. Illustration of the CTR Measurement on a Posterior-Anterior (PA) Chest Radiograph. This figure shows the method of determining the CTR, highlighting the greatest transverse dimensions of the heart (A+B) and chest cavity (C). The CTR is calculated as the sum of the heart’s transverse dimensions (A+B) divided by that of the chest cavity’s transverse dimension (C), providing a quantitative assessment of heart size relative to the chest cavity.
