# Supplementary material for: Correlations of sST2 and Gal-3 with Cardiothoracic Ratio in Patients with Chronic Kidney Disease
Source: Biomedicines. 2024 Apr 3;12(4):791. doi: 10.3390/biomedicines12040791 (PMC11048335; doi:10.3390/biomedicines12040791)
Supplement: Supplementary file 1 [file biomedicines-12-00791-s001.zip › biomedicines-2917265-supplementary.pdf]

Supplementary Materials:

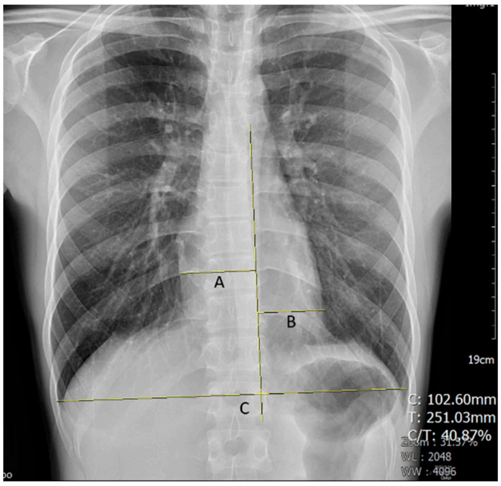

Figure S1. Illustration of the CTR Measurement on a Posterior-Anterior (PA) Chest Radiograph. This figure shows the method of determining the CTR, highlighting the greatest transverse dimensions of the heart (A + B) and chest cavity (C). The CTR is calculated as the sum of the heart's transverse dimensions (A + B) divided by that of the chest cavity's transverse dimension (C), providing a quantitative assessment of heart size relative to the chest cavity.
